# Propane-1,3-diyl bis­(4-amino­benzoate)

**DOI:** 10.1107/S1600536810026024

**Published:** 2010-07-07

**Authors:** Muhammad Raza Shah, Seik Weng Ng

**Affiliations:** a H. E. J. Research Institute of Chemistry, International Center for Chemical and Biological Sciences, University of Karachi, Karachi 75270, Pakistan; b Department of Chemistry, University of Malaya, 50603 Kuala Lumpur, Malaysia

## Abstract

Mol­ecules of the title compound, C_17_H_18_N_2_O_4_, lie on a twofold rotation axis that passes through the central methyl­ene C atom. The mol­ecules adopt a ‘V’ shape and the trimethyl­ene unit assumes a *gauche*–*gauche* conformation. The amino N atom shows a nonplanar coordination. Adjacent mol­ecules are connected by N—H⋯O hydrogen bonds into chains running along [001]. Furthermore, N—H⋯N hydrogen bonds connect these chains into a three-dimensional network.

## Related literature

For the crystal structure of 1,3-propandiyl-bis­(benzoate), see: Pérez & Brisse (1977 ▶).
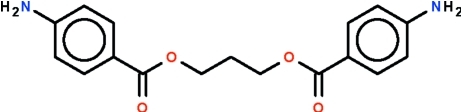


## Experimental

### 

#### Crystal data

C_17_H_18_N_2_O_4_
*M* *_r_* = 314.33
Monoclinic, 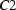

*a* = 23.725 (5) Å
*b* = 4.5109 (9) Å
*c* = 8.2171 (17) Å
β = 107.173 (3)°
*V* = 840.2 (3) Å^3^
*Z* = 2
Mo *K*α radiation
μ = 0.09 mm^−1^
*T* = 100 K
0.35 × 0.35 × 0.02 mm

#### Data collection

Bruker SMART APEX diffractometer
3936 measured reflections
1082 independent reflections
788 reflections with *I* > 2σ(*I*)
*R*_int_ = 0.090

#### Refinement

*R*[*F* ^2^ > 2σ(*F* ^2^)] = 0.043
*wR*(*F* ^2^) = 0.109
*S* = 0.96
1082 reflections
113 parameters
3 restraints
H atoms treated by a mixture of independent and constrained refinement
Δρ_max_ = 0.24 e Å^−3^
Δρ_min_ = −0.24 e Å^−3^

### 

Data collection: *APEX2* (Bruker, 2009 ▶); cell refinement: *SAINT* (Bruker, 2009 ▶); data reduction: *SAINT*; program(s) used to solve structure: *SHELXS97* (Sheldrick, 2008 ▶); program(s) used to refine structure: *SHELXL97* (Sheldrick, 2008 ▶); molecular graphics: *X-SEED* (Barbour, 2001 ▶); software used to prepare material for publication: *publCIF* (Westrip, 2010 ▶).

## Supplementary Material

Crystal structure: contains datablocks global, I. DOI: 10.1107/S1600536810026024/bt5289sup1.cif

Structure factors: contains datablocks I. DOI: 10.1107/S1600536810026024/bt5289Isup2.hkl

Additional supplementary materials:  crystallographic information; 3D view; checkCIF report

## Figures and Tables

**Table 1:** Hydrogen-bond geometry (Å, °)

*D*—H⋯*A*	*D*—H	H⋯*A*	*D*⋯*A*	*D*—H⋯*A*
N1—H11⋯O2^i^	0.86 (1)	2.15 (2)	2.958 (3)	157 (5)
N1—H12⋯N1^ii^	0.86 (1)	2.25 (1)	3.104 (3)	169 (2)

Symmetry codes: (i) 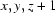; (ii) 
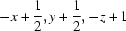
.

**Fig. 1.**
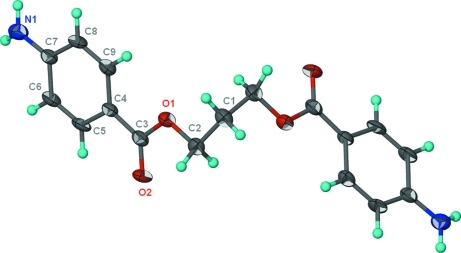
Anisotropic displacement ellipsoid plot (Barbour, 2001) of C17H18N2O4 at the 50% probability level; hydrogen atoms are drawn as spheres of arbitrary radius.

## supplementary crystallographic information

### Comment

The chemical is a commercially available chemical that should be compable of condensing with carbonyl compounds to yield Schff bases; its special feature is its trimethylene portion, which assumes a *V* shape. The C_17_H_18_N_2_O_4_ molecule (Scheme I) lies on a twofold rotation axis that passes through the central methylene carbon atom; this symmetry element relates one 4-aminobenzoate unit to the other. The molecule assumes a *V* shape and the trimethylene portion assumes a *gauche*–*gauche* conformation. The amino nitrogen atom shows non-planar coordination (Fig. 1). Adjacent molecules are connected by N–H···O and N–H···N shydrogen bonds to form a three-dimensional network.

### Experimental

The compound was returned unchanged but in a crystalline form in an unsuccessful condensation with *o*-vanillin in ethanol medium.

### Refinement

Carbon-bound H-atoms were placed in calculated positions [C–H 0.95–0.99 Å, *U*(H) 1.2*U*(C)] and were included in the refinement in the riding model approximation. The amino H-atoms were located in a difference Fourier map, and were refined isotropically with a distance restraint of N–H 0.86±0.01 Å. 822 Friedel pairs were merged.

### Crystal data

**Table:**

C_17_H_18_N_2_O_4_	*F*(000) = 332
*M**_r_* = 314.33	*D*_x_ = 1.242 Mg m^−^^3^
Monoclinic, *C*2	Mo *K*α radiation, λ = 0.71073 Å
Hall symbol: C 2y	Cell parameters from 915 reflections
*a* = 23.725 (5) Å	θ = 2.6–26.8°
*b* = 4.5109 (9) Å	µ = 0.09 mm^−^^1^
*c* = 8.2171 (17) Å	*T* = 100 K
β = 107.173 (3)°	Plate, yellow
*V* = 840.2 (3) Å^3^	0.35 × 0.35 × 0.02 mm
*Z* = 2	

### Data collection

**Table:**

Bruker SMART APEX diffractometer	788 reflections with *I* > 2σ(*I*)
Radiation source: fine-focus sealed tube	*R*_int_ = 0.090
graphite	θ_max_ = 27.5°, θ_min_ = 1.8°
ω scans	*h* = −30→29
3936 measured reflections	*k* = −5→5
1082 independent reflections	*l* = −10→10

### Refinement

**Table:**

Refinement on *F*^2^	Primary atom site location: structure-invariant direct methods
Least-squares matrix: full	Secondary atom site location: difference Fourier map
*R*[*F*^2^ > 2σ(*F*^2^)] = 0.043	Hydrogen site location: inferred from neighbouring sites
*wR*(*F*^2^) = 0.109	H atoms treated by a mixture of independent and constrained refinement
*S* = 0.96	*w* = 1/[σ^2^(*F*_o_^2^) + (0.0523*P*)^2^] where *P* = (*F*_o_^2^ + 2*F*_c_^2^)/3
1082 reflections	(Δ/σ)_max_ = 0.001
113 parameters	Δρ_max_ = 0.24 e Å^−^^3^
3 restraints	Δρ_min_ = −0.24 e Å^−^^3^

### Fractional atomic coordinates and isotropic or equivalent isotropic displacement parameters (Å^2^)

**Table:**

	*x*	*y*	*z*	*U*_iso_*/*U*_eq_	Occ. (<1)
O1	0.42724 (7)	0.4987 (4)	0.0488 (2)	0.0262 (5)	
O2	0.35969 (8)	0.7580 (5)	−0.1476 (2)	0.0304 (5)	
N1	0.29701 (11)	1.0783 (6)	0.5367 (3)	0.0298 (6)	
C1	0.5000	0.1862 (10)	0.0000	0.0279 (10)	
H1A	0.5095	0.0569	−0.0857	0.033*	0.50
H1B	0.4905	0.0569	0.0857	0.033*	0.50
C2	0.44676 (11)	0.3705 (7)	−0.0862 (3)	0.0263 (7)	
H2A	0.4572	0.5279	−0.1562	0.032*	
H2B	0.4153	0.2454	−0.1609	0.032*	
C3	0.38135 (11)	0.6876 (7)	0.0010 (3)	0.0244 (7)	
C4	0.36179 (11)	0.7924 (7)	0.1432 (3)	0.0228 (6)	
C5	0.31600 (11)	0.9969 (7)	0.1134 (3)	0.0260 (7)	
H5	0.2991	1.0721	0.0016	0.031*	
C6	0.29486 (12)	1.0917 (7)	0.2423 (3)	0.0286 (7)	
H6	0.2631	1.2289	0.2187	0.034*	
C7	0.31969 (11)	0.9879 (7)	0.4094 (3)	0.0252 (7)	
C8	0.36569 (11)	0.7851 (8)	0.4393 (3)	0.0295 (7)	
H8	0.3830	0.7123	0.5514	0.035*	
C9	0.38646 (11)	0.6885 (8)	0.3103 (3)	0.0282 (7)	
H9	0.4179	0.5495	0.3338	0.034*	
H11	0.3137 (17)	1.032 (12)	0.641 (2)	0.098 (16)*	
H12	0.2748 (10)	1.233 (4)	0.519 (3)	0.025 (8)*	

### Atomic displacement parameters (Å^2^)

**Table:**

	*U*^11^	*U*^22^	*U*^33^	*U*^12^	*U*^13^	*U*^23^
O1	0.0204 (9)	0.0351 (12)	0.0206 (9)	0.0041 (10)	0.0021 (7)	0.0031 (9)
O2	0.0239 (10)	0.0453 (13)	0.0165 (9)	0.0042 (10)	−0.0024 (7)	0.0029 (10)
N1	0.0272 (14)	0.0364 (17)	0.0227 (13)	0.0056 (12)	0.0025 (11)	0.0016 (12)
C1	0.021 (2)	0.030 (3)	0.029 (2)	0.000	0.0034 (16)	0.000
C2	0.0205 (13)	0.0329 (17)	0.0243 (14)	−0.0040 (13)	0.0046 (11)	−0.0024 (13)
C3	0.0172 (13)	0.0302 (17)	0.0224 (13)	−0.0048 (13)	0.0005 (11)	0.0009 (13)
C4	0.0163 (12)	0.0304 (17)	0.0183 (12)	−0.0025 (12)	−0.0001 (10)	0.0023 (11)
C5	0.0190 (13)	0.0322 (17)	0.0193 (13)	−0.0007 (14)	−0.0058 (11)	0.0043 (13)
C6	0.0191 (14)	0.0368 (19)	0.0236 (14)	0.0029 (13)	−0.0031 (12)	0.0038 (13)
C7	0.0186 (13)	0.0335 (18)	0.0202 (13)	−0.0060 (14)	0.0006 (10)	0.0005 (13)
C8	0.0218 (14)	0.043 (2)	0.0179 (13)	0.0039 (14)	−0.0026 (11)	0.0090 (14)
C9	0.0181 (14)	0.0397 (19)	0.0237 (14)	0.0030 (14)	0.0012 (11)	0.0050 (14)

### Geometric parameters (Å, °)

**Table:**

O1—C3	1.347 (3)	C3—C4	1.457 (4)
O1—C2	1.444 (3)	C4—C5	1.391 (4)
O2—C3	1.219 (3)	C4—C9	1.405 (3)
N1—C7	1.372 (3)	C5—C6	1.368 (4)
N1—H11	0.858 (10)	C5—H5	0.9500
N1—H12	0.861 (10)	C6—C7	1.405 (4)
C1—C2	1.503 (4)	C6—H6	0.9500
C1—C2^i^	1.503 (4)	C7—C8	1.389 (4)
C1—H1A	0.9900	C8—C9	1.365 (4)
C1—H1B	0.9900	C8—H8	0.9500
C2—H2A	0.9900	C9—H9	0.9500
C2—H2B	0.9900		
			
C3—O1—C2	116.39 (19)	C5—C4—C9	118.1 (2)
C7—N1—H11	121 (3)	C5—C4—C3	119.4 (2)
C7—N1—H12	118.1 (19)	C9—C4—C3	122.4 (2)
H11—N1—H12	116 (4)	C6—C5—C4	121.1 (2)
C2—C1—C2^i^	112.9 (4)	C6—C5—H5	119.4
C2—C1—H1A	109.0	C4—C5—H5	119.4
C2^i^—C1—H1A	109.0	C5—C6—C7	120.6 (3)
C2—C1—H1B	109.0	C5—C6—H6	119.7
C2^i^—C1—H1B	109.0	C7—C6—H6	119.7
H1A—C1—H1B	107.8	N1—C7—C8	121.7 (2)
O1—C2—C1	105.94 (18)	N1—C7—C6	120.0 (3)
O1—C2—H2A	110.5	C8—C7—C6	118.2 (2)
C1—C2—H2A	110.5	C9—C8—C7	121.2 (3)
O1—C2—H2B	110.5	C9—C8—H8	119.4
C1—C2—H2B	110.5	C7—C8—H8	119.4
H2A—C2—H2B	108.7	C8—C9—C4	120.7 (3)
O2—C3—O1	121.5 (2)	C8—C9—H9	119.7
O2—C3—C4	125.4 (3)	C4—C9—H9	119.7
O1—C3—C4	113.1 (2)		
			
C3—O1—C2—C1	176.5 (2)	C3—C4—C5—C6	177.4 (3)
C2^i^—C1—C2—O1	−71.92 (18)	C4—C5—C6—C7	1.1 (4)
C2—O1—C3—O2	−3.8 (4)	C5—C6—C7—N1	−178.2 (3)
C2—O1—C3—C4	176.2 (2)	C5—C6—C7—C8	−0.6 (4)
O2—C3—C4—C5	−2.2 (4)	N1—C7—C8—C9	177.6 (3)
O1—C3—C4—C5	177.8 (3)	C6—C7—C8—C9	0.1 (5)
O2—C3—C4—C9	176.1 (3)	C7—C8—C9—C4	0.1 (5)
O1—C3—C4—C9	−4.0 (4)	C5—C4—C9—C8	0.3 (4)
C9—C4—C5—C6	−0.9 (4)	C3—C4—C9—C8	−177.9 (3)

Symmetry codes: (i) −*x*+1, *y*, −*z*.

### Hydrogen-bond geometry (Å, °)

**Table:**

*D*—H···*A*	*D*—H	H···*A*	*D*···*A*	*D*—H···*A*
N1—H11···O2^ii^	0.86 (1)	2.15 (2)	2.958 (3)	157 (5)
N1—H12···N1^iii^	0.86 (1)	2.25 (1)	3.104 (3)	169 (2)

Symmetry codes: (ii) *x*, *y*, *z*+1; (iii) −*x*+1/2, *y*+1/2, −*z*+1.
